# The Role of Glyceraldehyde-3-Phosphate Dehydrogenases in NADPH Supply in the Oleaginous Filamentous Fungus *Mortierella alpina*

**DOI:** 10.3389/fmicb.2020.00818

**Published:** 2020-04-28

**Authors:** Shunxian Wang, Haiqin Chen, Xin Tang, Hao Zhang, Guangfei Hao, Wei Chen, Yong Q. Chen

**Affiliations:** ^1^State Key Laboratory of Food Science and Technology, Jiangnan University, Wuxi, China; ^2^School of Food Science and Technology, Jiangnan University, Wuxi, China; ^3^National Engineering Research Center for Functional Food, Jiangnan University, Wuxi, China; ^4^Wuxi Translational Medicine Research Center and Jiangsu Translational Medicine Research Institute Wuxi Branch, Wuxi, China; ^5^Beijing Innovation Centre of Food Nutrition and Human Health, Beijing Technology and Business University, Beijing, China; ^6^Department of Cancer Biology, Wake Forest School of Medicine, Winston-Salem, NC, United States

**Keywords:** glyceraldehyde-3-phosphate dehydrogenase, *Mortierella alpina*, NADPH supply, metabolic analysis, lipid biosynthesis

## Abstract

Glyceraldehyde-3-phosphate dehydrogenase (GAPDH) is a highly conserved enzyme within the glycolytic pathway. GAPDH catalyzes the transformation of glyceraldehyde 3-phosphate to glycerate-1, 3-biphosphate, a process accompanied by the production of NADH. Its role in the NADPH production system of the oleaginous filamentous fungus *Mortierella alpina* was explored. Two copies of genes encoding GAPDH were characterized, then endogenously overexpressed and silenced through *Agrobacterium tumefaciens*-mediated transformation methods. The results showed that the lipid content of the overexpression strain, MA-GAPDH1, increased by around 13%. RNA interference of GAPDH1 and GAPDH2 (MA-RGAPDH1 and MA-RGAPDH2) greatly reduced the biomass of the fungus. The lipid content of MA-RGAPDH2 was found to be about 23% higher than that of the control. Both of the lipid-increasing transformants showed a higher NADPH/NADP ratio. Analysis of metabolite and enzyme expression levels revealed that the increased lipid content of MA-GAPDH1 was due to enhanced flux of glyceraldehyde-3-phosphate to glycerate-1, 3-biphosphate. MA-RGAPDH2 was found to strengthen the metabolic flux of dihydroxyacetone phosphate to glycerol-3-phosphate. Thus, GAPDH1 contributes to NADPH supply and lipid accumulation in *M. alpina*, and has a distinct role from GAPDH2.

## Introduction

*Mortierella alpina* is an oleaginous filamentous fungus with a strong ability to accumulate polyunsaturated fatty acids (PUFAs) and has been used in industrial production of arachidonic acids (ARA) (Tsunehiro et al., 2001; Higashiyama et al., 2002; Dyal and Narine, 2005). Great efforts have been made to improve its lipid yield, production and productivity (Koike et al., 2001; Sakuradani et al., 2009; Hao et al., 2016). A traditional and effective way to achieve this is increasing the supply of NADPH, which is indispensable during lipid biosynthesis. Known enzymes involved in this process include malic enzyme, glucose-6-phosphate dehydrogenase and 6-phosphogluconic dehydrogenase from the phosphate pentose pathway and isocitrate dehydrogenase (Hao et al., 2014a, b, 2016). A clear understanding of all NADPH sources for lipid biosynthesis is important for expounding the lipid biosynthesis process. Studies aimed to identify enzymes that contribute to NADPH production for lipid biosynthesis are necessary.

Glyceraldehyde-3-phosphate dehydrogenase (GAPDH) is one such enzyme. It catalyzes the conversion of glyceraldehyde 3-phosphate to glycerate-1, 3-biphosphate, and results in production of NADH. The NADH produced from the reaction catalyzed by GAPDH can be transformed into NADPH in the cytoplasm through the pyruvate-oxaloacetate-malate cycle (POM cycle). The *gapdh* is traditionally used as a conserved gene for species identification (Ghuffar et al., 2018; Huang et al., 2018; Torres-Calzada et al., 2018). Its promoter is highly effective for heterologous protein expression in microorganisms (Madhavan et al., 2017; Prielhofer et al., 2017; Liu et al., 2018). In recent years, GAPDH has been considered to be a moonlighting protein, with multiple known functions including transcriptional activation, signal transduction, cellular apoptosis and abiotic stress (Tristan et al., 2011; Nicholls et al., 2012; Zhang et al., 2017). Ratledge demonstrated its potential role in NADPH production in oleaginous microorganisms in 2014. According to his stoichiometric analysis, GAPDH could provide 1.16 moles of NADPH among the 16 moles of NADPH needed for synthesizing 1 mole of C18-fatty acyl-CoA (Ratledge, 2014). Overexpression a gene encoding the NAD+-dependent GAPDH together with three other genes (encoding malate dehydrogenase, pyruvate-formate lyase, and formate dehydrogenase) led to a 1.2-fold increase in intracellular NADH pool in the bacteria *Shewanella oneidensis* (Li et al., 2018). Thus, it is necessary to verify the role of GADPH in NADPH generation in oleaginous microorganisms.

The aim of this study is to explore the role of GAPDH in NADPH production system in oleaginous filamentous fungus *M. alpina*. Homologous overexpression and RNA interference technology were used. Metabolite extraction was also employed to obtain insights on the metabolic flux changes in the engineered strains. The role of GAPDH as NADPH supplier in the oleaginous microorganisms was only theoretically discussed before. Exploring new NAPDH suppliers is beneficial to elucidate the lipid biosynthesis mechanisms in oleaginous microorganisms.

## Materials and Methods

### Strains and Medium

*E. coli* TOP10 was used for plasmid construction and maintenance. *Agrobacterium tumefaciens* AGL-1 was used for transforming *M. alpina*. Uracil *M. alpina* MAU1 (CGMCC No. 8414) was used as the initial strain. The binary plasmid pBIG2-ura5-ITs (United States Patent US9982269B2) was used as the vector. Wild type *M. alpina* ATCC32222 was used as the control group.

LB medium, YEP medium (10 g/L yeast extract, 10 g/L tryptone, 5 g/L NaCl) and broth medium (20 g/L glucose, 5 g/L yeast extract, 1 g/L KH_2_PO_4_, 0.25 g/L MgSO_4_, 10 g/L KNO_3_) were used for culturing *E. coli* TOP10, *A. tumefaciens* AGL-1 and *M. alpina* ATCC32222, respectively. Specially, broth medium with 50 g/L glucose was used when operating the flask fermentation. MM medium (1. 74 g/L K_2_HPO_4_, 1.37 g/L KH_2_PO_4_, 0.146 g/L NaCl, 0.49 g/L MgSO_4_⋅7H_2_O, 0.078 g/L CaCl_2_, 0.0025 g/L FeSO_4_⋅7H_2_O, 0.53 g/L (NH_4_)_2_SO_4_, 1.8 g/L glucose, pH 7.0), IM medium (MM containing 0.5% (w/v) glycerol, 200 μM acetosyringone, 40 mM 2-(*N*-morpholino) ethane sulfonic acid (MES), pH 5.3) and SC medium (SC medium was used as a uracil-free synthetic medium, which contained 5 g of yeast nitrogen base without amino acids and ammonium sulfate, 1.7 g/L of (NH_4_)_2_SO_4_, 20 g/L of glucose, 20 mg/L of adenine, 30 mg/L of tyrosine, 1 mg/L of methionine, 2 mg/L of arginine, 2 mg/L of histidine, 4 mg/L of lysine, 4 mg/L of tryptophan, 5 mg/L of threonine, 6 mg/L of isoleucine, 6 mg/L of leucine, and 6 mg/L of phenylalanine) were as previously reported (Ando et al., 2009; Chen et al., 2015) and used for *A. tumefaciens* mediated transformation of *M. alpina*.

### Plasmid Construction

Fragments used for overexpression were produced using polymerase chain reactions (PCR) of a cDNA library of wild *M. alpina* ATCC32222 (genome information can be assessed by BioProject No PRJNA41211), and constructed through RT-qPCR (Thermo Fisher, United States) of total RNA extracted using Trizol reagent (Invitrogen, United States) (Hao et al., 2014b). Primers used in the manuscript were designed through DNAman 7.0 (Online Resource Table 1). The length of the designed primers was 21 bp. The evaluated points of primer pairs for GAPDH1 and GAPDH2 exported were 675 and 672, respectively. Then the enzymatic sites HindIII and SacI and corresponding protection bases were added. The designed primers were synthesized by Sunnybio (Shanghai, China). HindIII, SacI and T4 ligase (Thermo Fisher, United States) were used for fragment digestion and plasmid construction. *E. coli* TOP10 and *A. tumefaciens* AGL-1 were used for plasmid storage. The RNA interference technology of *M. alpina* established in 2015 by our team was used in this manuscript as before (Chen et al., 2015) (Supplementary Figure 1), with the plasmid being constructed by General Biosystems (Anhui) Co. Ltd. (Anhui, China).

### *A. tumefaciens* Mediated Transformation

The process of *A. tumefaciens*-mediated transformation was as per standard protocol (Hao et al., 2014b). Transformed *A. tumefaciens* containing the binary plasmid was cultured in YEP solid medium, YEP liquid medium, MM medium successively. The appropriate concentration of spores and *A. tumefaciens* were co-cultured together following the induction in IM liquid medium. Minor changes to the protocol were made, as per the following: germinating spores instead of fresh spores were used as co-cultivation materials, and 16°C was used as co-cultivation temperature for 24–48 h before 23°C cultivation.

### Flask Fermentation

Broth medium with 50 g/L glucose was used for flask fermentation. 100 μL 10^6^/mL spores were incubated in broth medium with 20 g/L glucose and cultured for two generations with a blender (TKA, Germany). The strains were subsequently cultured at 28°C for 7 days with shaking (200 rpm) in a 250 mL flask.

### Fatty Acid Extraction and Analysis

The fatty acid extraction method was as per previous report (Hao et al., 2015). 50 mg mycelium was destroyed using physical stimuli through alternating temperature and chemical treatment by hydrochloric acid. Total lipids were extracted by treatment with *n*-hexane and trichloromethane, then esterified using methanol-HCl. Extracted fatty acid methyl ester samples were analyzed by GC–MS (GC-2010 Plus; MS-QP2010 Ultra, Shimadzu, Co., Kyoto, Japan) as per standard protocol (Wang et al., 2011).

### Transcriptional Level Analysis

The expression level of GAPDH was detected using RT-qPCR. Total RNA was extracted and reversely transcribed into DNA as per PrimeScript RT reagent kit’s instructions (Thermo Fisher, United States). 1 μL DNA template was added to the reaction mixture consisting of 10 μL of SYBR green PCR master mix, 8 μL of ddH_2_O and 0.5 μL of primer pairs. The 18S rRNA gene was used as internal gene control. The procedure on ABI Prism 7900 sequence detection system (Foster City, CA, United States) was as follows: 50°C for 2 min, 95°C for 10 min, and 40 cycles of amplification at 95°C for 15 s and 60°C for 30 s.

### Enzyme Activity Determination

Enzyme activity was determined as per the GAPDH activity assay kit’s protocol (Biovision, United States). 10 mg mycelium was homogenized with 100 μL GAPDH assay buffer. 10 μL extracted buffer was added into the 96-well plate with 40 μL GAPDH assay buffer. A NADH standard curve was made based on a series of wells with concentrations of 0, 2.5, 5.0, 7.5, 10, and 12.5 nmol/well. A background control without GAPDH substrate was also added. All samples and standards were run in triplicate. 50 μL reaction mix was added to each well. The plate was measured at 450 nm in kinetic mode for 10–60 min at 37°C (T1 and T2). All samples and standards were run in triplicate. The NADH stand curve was read at the end point. Subtract the background control OD value from all sample readings. We calculated the ΔOD for the GAPDH activity of the test sample as follows:

(1) ΔOD=A-2A1

The B nmol of NADPH generated by GAPDH activity during the reaction time (ΔT, Eq. 2) can be achieved using the ΔOD to the NADH standard curve.

(2) ΔT=T-2T1

Then the sample GAPDH activity was calculated using the following equation:

Sample⁢GAPDH⁢activity

(3) =B/(Δ⁢T*V)*D=nmol/min/μ⁢l=mU/μ⁢l=U/ml

B is the NADH amount from standard curve (nmol). ΔT is the reaction time (min.) V is the sample volume added into the reaction well (μl). D is dilution factor.

### NADP+ and NADPH Level Determination

The concentrations of NADP**+** and NADPH were determined as per the protocol of the NADP+ /NADPH Quantification Colorimetric kit (Sigma-Aldrich, United States). An NADPH standard curve was generated using colorimetric detection along with the experiment. Each well in the 96-well plate required 50 mg tissue. NADP+ /NADPH extraction buffer, NADP+ cycling buffer and NADP+ cycling enzyme mix were needed for total NADP+ and NADPH detection. All samples and standards were run in triplicate.

### Metabolite Extraction and Analysis

The freeze-dried biomass was prepared, and the intracellular metabolites were extracted using methanol: water (1:1, v/v) as per previous research (Lu et al., 2019). The vacuum-dried samples were resuspended in MeOX-pyridine and MSTFA with 1% TMCS. Then, the extracted compounds were detected using GC-MS. Peak extraction, retention time adjustment, peak alignment, deconvolution analysis, and identification were performed using MS-DIAL 3.70 equipped with a DB_FiehnBinbase-FiehnRI database.

### Statistical Treatment

The achieved data were analyzed with GraphPad Prism 6.0. Ordinary one-way ANOVA and Dunnett’s multiple comparisons test were used to assert significant differences. The error bars represent standard deviations. One asterisk represents *p* < 0.05. Two asterisks represent *p* < 0.01. Three asterisks represent *p* < 0.001.

## Results

### Identification of *gapdh* in *M. alpina* and Corresponding Bioinformatic Analysis

**FIGURE 1:**
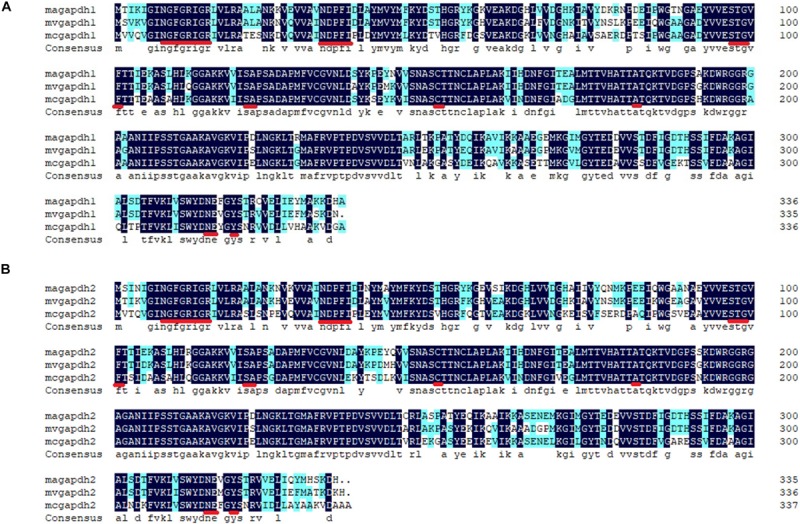
Alignments of the predicted genes with verified genes of other species. **(A)** alignments of GAPDH1; **(B)** alignments of GAPDH2. The alignments were carried out by the software DNAman. Sequence of other GAPDH amino acids were downloaded from website of NCBI. Magapdh1, *Mortierella alpina* ATCC32222, GI: MN417267; mvgapdh1, *Mortierella verticillata* NRRL6337, GI: 672823544; mcgapdh1, *Mucor circinelloides f. circinelloides* 1006PhL, GI: 511008895; magapdh2, *M. alpina* ATCC32222, GI: MN417268; mvgapdh2, *Mortierella verticillata* NRRL6337, GI: 672823261; mcgapdh2, *Mucor circinelloides f. circinelloides* 1006PhL, GI: 511007306.

Two GAPDH genes of 1011 and 1008 bp [named *gapdh1* (MN417267 in GenBank) and *gapdh2* (MN417268 in GenBank), respectively] in length were recognized in *M. alpina*. Multiple sequence alignments were carried out using corresponding GAPDH amino acid sequences from other species (Figure 1). Results showed that the GAPDH1 and GAPDH2 of *M. alpina* shared 87 and 84% similarities with other known GAPDH proteins, respectively. Based on the X-ray structure of lobster GAPDH (Moras et al., 1975), the 24 residues (—NGFGRIGR—NDPFI—STG—F—SA—C—A—NE—Y—) with which the NAD+ interacted in that structure were found to be identical in GAPDH1 and GAPDH2 were marked with red underline in Figure 1. Corresponding protein structures were predicted by Swiss Model^1^. Both genes were predicted to be homo-tetramers, and the ligands were four NAD+ molecules. Subcellular localization of the two genes was also predicted using Cell PLoc 2.0. Results showed that both GAPDH are likely located in the cytoplasm or mitochondria. Since the glycolysis process before producing the pyruvate usually happens in the cytoplasm, it is no wonder that the possible location of GAPDH is in cytoplasm. The predicted mitochondrial localization is interesting, as it indicates that GAPDH may participate in other biological process. The above information indicates that the identified genes in *M. alpina* encode GAPDH and can produce NADH in the cytoplasm.

### Transcriptional Analysis of *gapdh1* and *gapdh2* Shows They Have Opposing Roles During Lipid Accumulation

**FIGURE 2:**
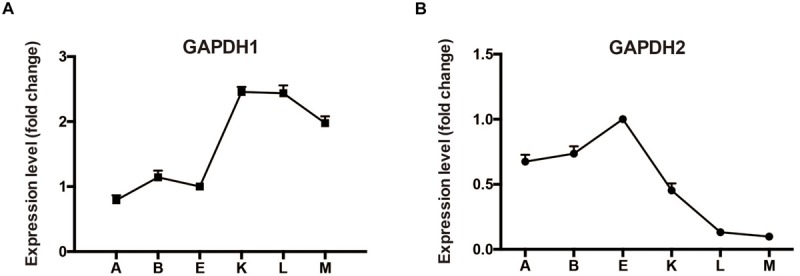
Expression level of GAPDH during batch fermentation **(A)** expression level change of GAPDH1; **(B)** expression level change of GAPDH2. *M. alpina* was cultured in a 5 L fermenter and samples were collected at time points before and after the nitrogen exhaustion (sample A: –12 h, B: –2 h, E: –30 min, K: +1 h, L: +12 h, and M: +48 h). Three replicates were achieved for the flask fermentation.

Typically, *M. alpina* has two distinctive growth phases during fermentation process (Chen et al., 2015). During the first phase when there are sufficient nutrients in the growth medium, the biomass increases stably. When nitrogen exhausts and glucose is present, growth of *M. alpina* ceases and the lipid begins to accumulate. Samples prior to nitrogen exhaustion, during nitrogen exhaustion and after nitrogen exhaustion were collected (sample A: -12 h, B: -2 h, E: -30 min, K: +1 h, L: +12 h, and M: +48 h) to determine the transcriptional level of *gapdh* during lipid biosynthesis in wild type *M. alpina* (Figure 2). Results showed the transcript level of *gapdh1* increased following nitrogen exhaustion and remained at a higher level during the lipid biosynthetic process. Conversely, the transcript level of *gapdh2* showed the opposite trend, indicating the two genes have opposite roles during the lipid accumulation process.

### Genetic Manipulation of *gapdh1* and *gapdh2* Affects the Biomass and Total Fatty Acid of *M. alpina* Through an Altered NADPH/NADP Ratio

**FIGURE 3:**
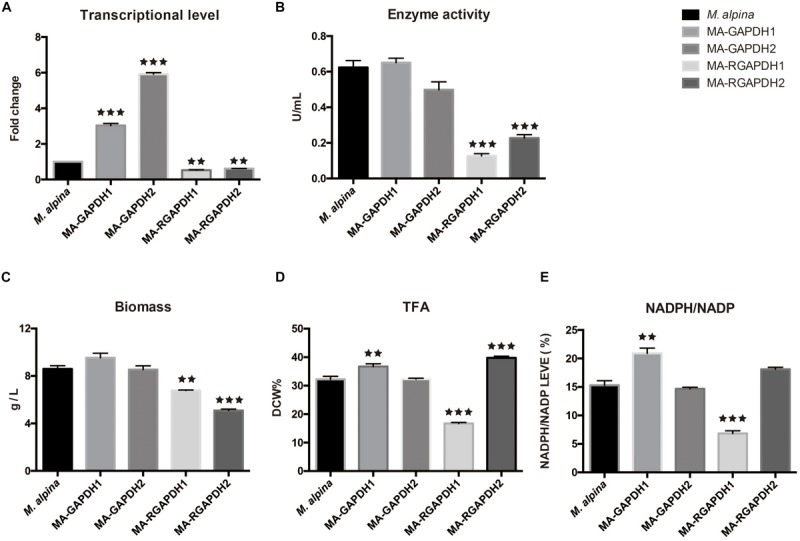
Effect of GAPDH1 and GAPDH2 on lipid biosynthesis **(A)** transcriptional level analysis; **(B)** enzyme activity analysis; **(C)** biomass analysis; **(D)** total fatty acid (TFA) analysis; **(E)** NADPH level analysis. ** represents *p* < 0.01, *** represents p < 0.001.

The two *gapdh* genes were cloned through high-fidelity PCR with c-DNA of the wild type *M. alpina* as substrate. The homologous overexpression plasmids and RNA interference plasmids were subsequently constructed. The genes were homologous overexpressed under control of the *M. alpine* histone H550 promoter that was modified from previous study (Mackenzie et al., 2000) and the *A. nidulans* trpC transcription terminator (trpCt) (Hao et al., 2014a) (Supplementary Figure 1). The T-DNA was successfully integrated into the genome of *M. alpina* using the ATMT method (Supplementary Figure 2). The engineered strains were cultured using flask fermentation for 7 days. Measurement of transcriptional level and enzyme activity was employed to confirm the successful overexpression (MA-GAPDH1 and MA-GAPDH2) or knockdown (MA-RGAPDH1 and MA-RGAPDH2) of the genes (Figures 3A,B). Notably, the enzyme activity of MA-RGAPDH1 and MA-RGAPDH2 in which the expression of *gapdh* genes were suppressed through RNA interference were greatly induced. The residual enzyme activity in the two strains is probably due to the uneliminated mRNAs due to the characteristic of RNA interference technology. However, the result with greatly reduced enzyme activity in the two knock-down strains can still offer reference for explanation the role of GAPDH.

As per the fermentation results (Figures 3C,D), the biomass and total fatty acid (TFA) of the MA-GAPDH1 increased approximately 10% while those of MA-GAPDH2 showed no change. The same tendency was also observed in the ratio of NADPH/NADP (Figure 3E). Overexpression of GAPDH2 didn’t make significant changes. The growth of the MA-RGAPDH1 and MA-RGAPDH2 strains were significantly impaired when these genes were silenced (Figure 3C). The TFA of MA-RGAPDH1 showed a 50% decrease while that of MA-RGAPDH2 showed a 23.22% increase (Figure 3D). The NADPH/NADP ratio of the two strains also followed a similar pattern (Figure 3E). This indicates that GAPDH1 contributes to NADPH production during lipid biosynthesis.

### Increased Lipid Content of Strains MA-GAPDH1 and MA-RGAPDH2 Is Due to Strengthening Flux of Different Branches of Fructose -1, 6-Biphosphate

**FIGURE 4:**
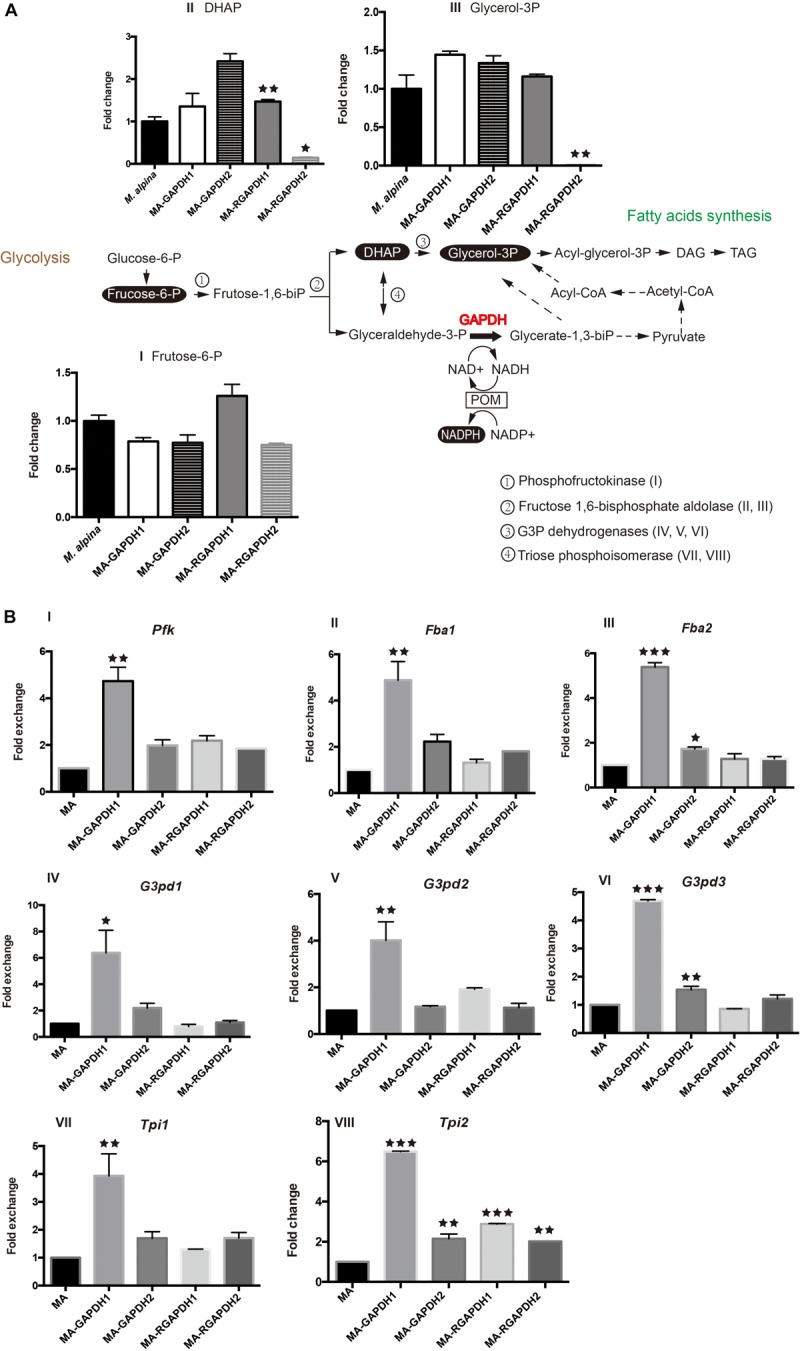
Metabolic analysis of the pathways the overexpression transformants and RNA interference transformants **(A)** substrate and product analysis of the GAPDH pathway; **(B)** transcription level of the selected genes. P represents the abbreviation of phosphate, glucose-6-P-glucose-6-phosphate, frucose-6-P-frucose-6-phosphate, frucose-1,6-biP-frucose-6-biphosphate, DHAP-dihydroxyacetone phosphate, glycerol 3P-glycerol-3-phosphate, acyl-glycerol-3P- acyl-glycerol-3-phosphate, DAG-diacylglycerol, TAG-triacylglycerol, glyceraldehyde-3-P- glyceraldehyde-3-phosphate, glycerate-1,3-biP-glycerate-1,3-biphosphate, POM-pyruvate/oxaloacetate/malate enzyme cycle, *Pfk*-phosphofructokinase, *Fba*-fructose-1, 6-biphosphate aldolase, *G3pd-*Glycerol-3-phosphate dehydrogenases, *Tpi*-triose phosphoisomerase. * represents *p* < 0.05, ** represents *p* < 0.01, *** represents *p* < 0.001.

Metabolic analysis of the engineered strains was carried out to investigate the changes of fructose-1, 6-biphosphate levels in the engineered strains. Notably, the transformation of dihydroxyacetone phosphate (DHAP) and glyceraldehyde-3-phosphate (glyceraldehyde-3-P) is reversible (Figure 4A, double-headed arrow). Thus, the expression levels of the genes (phosphofructokinase, *Pfk*; fructose-1, 6-biphosphate aldolase, *Fba*; glycerol-3-phosphate dehydrogenases, *G3pd*; triose phosphoisomerase, *Tpi*) involved in these reactions were also tested (Figure 4A). The used metabolite analysis method was unable to identify fructose-1, 6-biphosphate, glyceraldehyde-3-P and glycerate-1, 3-biphosphate (glycerate-1, 3-biP), thus, fructose-6-phosphate (fructose-6-P), DHAP and glycerol-3-phosphate (glycerol-3P) were chosen as substitutes.

MA-GAPDH1 and MA-GAPDH2 exhibited similar decreases in fructose-6-P, indicating enhanced catalysis by GAPDH due to the overexpression of the genes (Figure 4AI). This is in accordance with the transcriptional levels of *Pfk* (Figure 4BI), *Fba*1 (Figure 4BII) and *Fba*2 (Figure 4BIII). The DHAP level in the MA-GAPDH1 strain was lower than that of MA-GAPDH2 (Figure 4AII), while the glycerol-3P level in the MA-GAPDH1 strain was higher (Figure 4AIII). Combined with the altered transcriptional levels of *Tpi1* and *Tpi2* (Figure 4BVII,VIII), GAPDH1 possesses a stronger catalyzing ability than GAPDH2. The enhanced lipid content of MA-GAPDH1 is due to the enhanced generation of glycerate-1, 3-biP from glyceraldehyde-3-P.

The MA-RGAPDH1 strain had higher fructose-6-P, DHAP and glycerol-3P levels than the control while those of the MA-RGAPDH2 strain were lower (Figure 4AI,II,III). The increased levels of fructose-6-P, DHAP and glycerol-3P in the MA-RGAPDH1 strain were caused by reduced catalysis due to the knockdown of GAPDH1. Theoretically, the same phenomena would also happen in the MA-RGAPDH2 strain. Unexpectedly, the content of fructose-6-P in MA-RGAPDH2 increased rather than decreased. The different changes of fructose-6-P between MA-RGAPDH1 and MA-RGAPDH2 further indicated the different role of GAPDH1 and GAPDH2 during the lipid biosynthesis process in *M. alpina* besides the transcriptional level changes showed in Figure 2. The DHAP and glycerol-3P levels of the MA-RGAPDH2 strain showed significant reductions (Figure 4AII,III). The transcriptional level of *Pfk*, *Fab1/Fab2*, *G3pd2/G3pd3* and *Tpi1/Tpi2* in the MA-RGAPDH1 and MA-RGAPDH2 strains both showed slightly higher levels than the control (Figure 4B). Thus, the strong reduction in DHAP and glycerol-3P levels in the MA-RGAPDH2 strain indicated that they were highly catabolized, indicating a strengthening flux from DHAP to glycerol-3P in the MA-RGAPDH2 strain.

Overall, the increased lipid content of the MA-GAPDH1 and MA-RGAPDH2 strains was due to the strengthening of different catalytic pathways. Overexpressing GAPDH1 promoted lipid biosynthesis through enhancing flux from glyceraldehyde-3-P to glycerate-1, 3-biP, providing NADPH and glycerol-3P for lipid biosynthesis. In MA-RGAPDH2 strains, the strengthened metabolic flux from DHAP to glycerol-3P increased the levels of glycerol-3P available for triacylglycerol synthesis, therefore, the greatly reduced glycerol-3P level in these cells (Figure 4AIII) was due to its usage as carbon skeleton for triacylglycerol synthesis.

### Proline and Tryptophan Showed Significant Increases in the MA-GAPDH1 and MA-RGAPDH2 Strains

**FIGURE 5:**
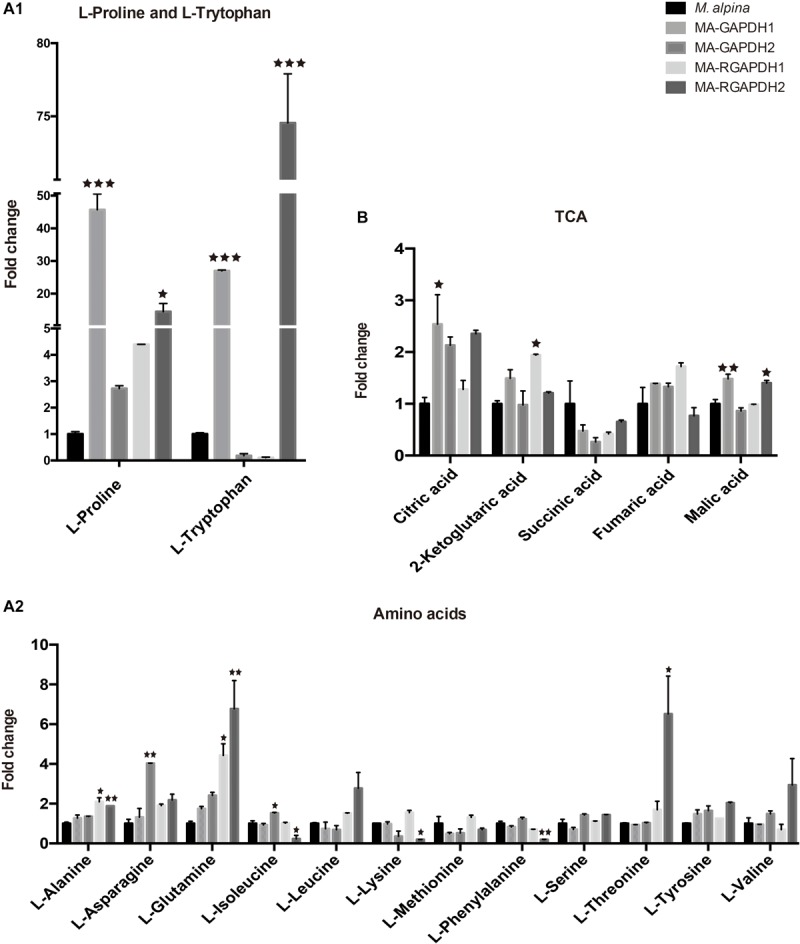
Metabolic analysis of the amino acids and TCA (tricarboxylic acid cycle) cycle of the engineered strains **(A1)** proline and tryptophan analysis; **(A2)** other amino acids analysis; **(B)** TCA cycle analysis. * represents *p* < 0.05, ** represents *p* < 0.01, *** represents *p* < 0.001.

Metabolite analysis of amino acids and TCA cycle was also carried out. Both MA-GAPDH1 and MA-RGAPDH2 strains showed increased TFA content as well as significantly increased proline and tryptophan levels. Results showed overexpressing GAPDH1 greatly increased proline and tryptophan levels by around 50- and 30-fold, respectively, while other amino acids didn’t show such great changes (Figure 5A1,A2). The same result was also observed in MA-RGAPDH2. A 2-fold enhancement in the citric acid level was seen in the MA-GAPDH1 and MA-RGAPDH2 strains, indicating enhanced TCA cycle flux (Figure 5B). The reason why the two amino acids showed obvious changes is unknown. The metabolic pathway of proline and tryptophan may have relationship with reactions that the GAPDH are involved in.

## Discussion

Lipid analysis further demonstrates that the two GAPDH genes exert different functions during lipid accumulation in *M. alpina*, which is in accordance with the changes in transcriptional levels. The greatly decreased biomass of the RNA interference strains (MA-RGAPDH1 and MA-RGAPDH2) demonstrates that the two genes play important roles in maintaining biomass. GAPDH1 contributes more to biomass formation than GAPDH2, according to these results. Although GAPDH1 can contribute to NADPH production, it does not make as much effect on fatty acid synthesis as either malic enzyme or the phosphate pentose pathway, which increase lipid content by 1.5-fold and 1.7-fold when being overexpressed in *M. alpina*, respectively (Hao et al., 2016). This result is in accordance with Ratledge’s stoichiometric analysis (Ratledge, 2014).

The different role of GAPDH1 and GAPDH2 in lipid biosynthesis in *M. alpina* is interesting, particularly the result of GAPDH2. As we mentioned in the introduction, the GAPDH2 might exhibit a role as a moonlighting protein. In *Saccharomyces cerevisiae*, the GAPDH2 was verified having interaction with the ubiquitously expressed glutathione peroxidases 3, which is one of the key anti-oxidant enzymes (Lee et al., 2011). The GAPDH2 of filamentous cyanobacteria is responsible for cell growth through photosynthetic carbon fixation and helps export carbohydrate from vegetative cells to the heterocysts (Valverde et al., 2001). The different roles of GAPDH1 and GAPDH2 are probably due to the different regulatory mechanisms. Evidence revealed that the regulation of GAPDH1 and GAPDH2 of *Drosophila* is different due to different organization of regulatory sequences (Sun et al., 1988b). A team found that URS-2, one of the regulatory regions, plays a key role in the developmental regulation of GAPDH2 in *Drosophila* (Sun et al., 1988a). Thus, different enzyme modifications of GAPDH1 and GAPDH2 also possibly exist in *M. alpina* and may explain their difference in lipid biosynthesis. A certain post-translation modification of GAPDH2 in *M. alpina* may explain the results of MA-GAPDH2, where the enzyme activity, NADPH/NADP ratio, lipid content didn’t change so much compared with control but not the transcriptional level (Figure 3). While, a different enzyme modification of GAPDH1 may exist and allow it to regulate the transcription of some genes (such as *Pfk* and *Fbas* in Figure 4B), resulting in greater transcriptional levels fold changes of *Pfk* and *Fbas* in MA-GAPDH1 than MA-GAPDH2 (Figure 4BI,II,III). The above hypotheses make sense to explain the difference of GAPDH1 and GAPDH2 in lipid biosynthesis in *M. alpina*.

The prominent changes in proline and tryptophan of the two transformants are also interesting. The specific reasons are unknown. Proline accumulation and very-long-chain fatty acid synthesis in *Arabidopsis thaliana* were shown sharing dual roles to help buffer cellular redox status while producing products useful for stress resistance, namely the compatible solute proline and cuticle lipids (Shinde et al., 2016). The above result of proline may also work in this way in *M. alpina* during the lipid fermentation process. Studies about specific relationship of tryptophan with lipid biosynthesis or reactions involved with GAPDH are not found. A gene encoding cytochrome P450 monooxygenase (*CYP79D63*) from *Erythroxylum coca* was heterologously expressed in *Saccharomyces cerevisiae.* The recombinant exclusively accepted tryptophan as substrate to synthesize aldoximes for stress defense (Luck et al., 2016). This research gives us a hint that the obviously enhanced tryptophan in *M. alpina* may also be the substrate for some chemicals useful for stress defense in the microorganism. The results about the prominent changes of proline and tryptophan during the lipid biosynthesis process in *M. alpina* provide interesting questions for the researchers in the field.

## Conclusion

This paper first experimentally verified the role of GAPDH in NADPH production in oleaginous microorganism. Two *gapdh* genes were recognized and then overexpressed and suppressed in filamentous fungus *M. alpina*. The transformants of the GAPDH1 overexpressed strain and GAPDH2 RNA interference strain showed both increased lipid content as well as ratio of NADPH. Further metabolic analysis showed the two kinds of transformants might have strengthened different metabolic flux. Overall, the GAPDH was first experimentally tested capable of contributing to NADPH supply for lipid accumulation in oleaginous microorganisms. This finding supplements a new member of the NADPH suppliers for lipid biosynthesis and helps elucidate the lipid biosynthesis mechanisms in oleaginous microorganism.

## Data Availability Statement

The datasets generated for this study can be found in the DNA sequence for gapdh1:MN417267 in NCBI, DNA sequence for gapdh2: MN417268 in NCBI.

## Author Contributions

SW curated the data, worked on the software, and wrote the original draft. HC, HZ, GH, and WC acquired funding. HZ and WC provided resources. XT supervised the study. HC, XT, and YC reviewed and edited the manuscript.

## Conflict of Interest

The authors declare that the research was conducted in the absence of any commercial or financial relationships that could be construed as a potential conflict of interest.

## Footnotes

1 https://swissmodel.expasy.org/interactive
