# Supplementary material for: The Role of Glyceraldehyde-3-Phosphate Dehydrogenases in NADPH Supply in the Oleaginous Filamentous Fungus Mortierella alpina
Source: Front Microbiol. 2020 Apr 28;11:818. doi: 10.3389/fmicb.2020.00818 (PMC7198782; doi:10.3389/fmicb.2020.00818)
Supplement: Supplementary file 1 [file Data_Sheet_1.PDF]

Supplementary Material

1 Supplementary Figures

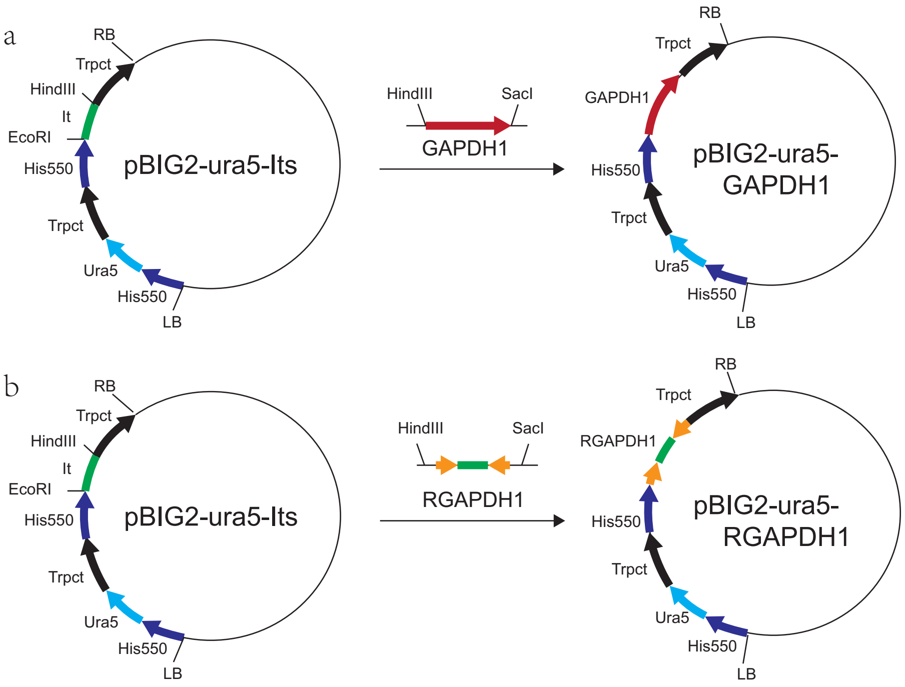

Supplementary Figure 1. Construction of binary plasmids for homologous overexpression and RNA interference a. demonstration of construction of binary plasmids overexpression of GAPDH1, GAPDH1 was amplified with PCR from cDNA then was digested by HindIII and EcoRI, then was ligated with the digested pBIG2-ura5-ITs by T4 ligase; b. demonstration of construction of binary plasmids RNA interference of GAPDH1, the 150bp homologous fragment was designed as forward

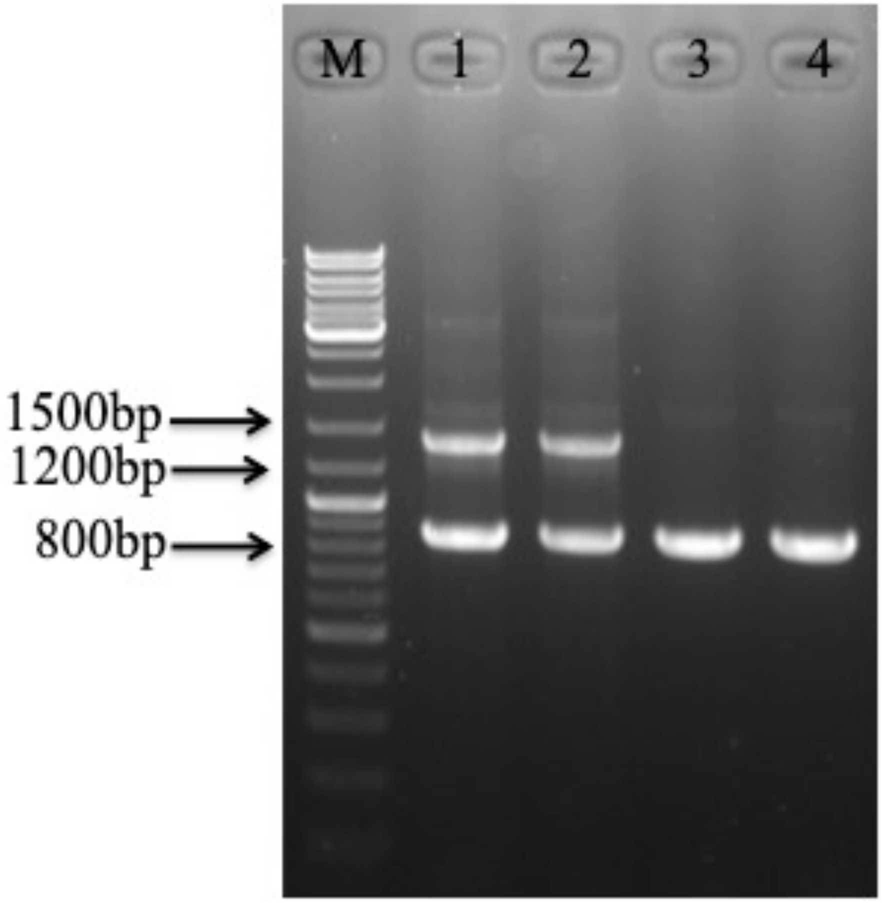

Supplementary Figure 2. Electrophoresis results of the overexpression transformants and RNA interference transformants The PCR system contained genome of the engineered strains as substrate and the general primers. 1. MA-GAPDH1; 2. MA-GAPDH2; 3. MA-RGAPDH1; 4. MA-RGAPDH2.
